# Network localization of regional homogeneity alterations in Parkinson’s disease

**DOI:** 10.3389/fnagi.2025.1607691

**Published:** 2025-05-19

**Authors:** Yuanying Song, Hucheng Yang, Siyu Gu, Yingling Zhu, ZhenYu Dai, Pinglei Pan, Xianxian Zhang

**Affiliations:** ^1^Department of Neurology, The Yancheng School of Clinical Medicine of Nanjing Medical University, Yancheng Third People’s Hospital, Yancheng, China; ^2^Department of Radiology, The Yancheng School of Clinical Medicine of Nanjing Medical University, Yancheng Third People’s Hospital, Yancheng, China; ^3^Department of Radiology, Binhai Maternal and Child Health Hospital, Yancheng, China; ^4^Education Department, The Yancheng School of Clinical Medicine of Nanjing Medical University, Yancheng Third People’s Hospital, Yancheng, China

**Keywords:** Parkinson’s disease, regional homogeneity, functional connectivity network mapping, network localization, resting-state functional MRI

## Abstract

**Background:**

Resting-state functional MRI (rs-fMRI) studies using regional homogeneity (ReHo) have identified localized functional changes in Parkinson’s disease (PD), but findings across studies exhibit considerable heterogeneity. The emerging network perspective suggests these disparate findings might reflect nodes within a single interconnected network. Functional Connectivity Network Mapping (FCNM) offers an approach to test this hypothesis.

**Methods:**

We conducted a systematic literature search (PubMed, Embase, Web of Science, CNKI, and Wanfang) for studies reporting whole-brain ReHo differences (PD vs. healthy controls). Resting-state fMRI data from the Human Connectome Project (HCP; *n* = 1,093) were analyzed using FCNM to map ReHo abnormalities in PD onto common functional brain networks. Robustness was assessed using 1 mm and 7 mm radii, and spatial overlap with canonical brain networks was quantified.

**Results:**

A total of 52 studies, comprising 72 datasets reporting ReHo differences between 2,052 PD patients and 1,401 healthy controls, were included in the analysis. The FCNM analysis identified a distributed PD-associated dysfunctional network. This network showed significant spatial overlap primarily with the visual (49.24%), somatomotor (32.35%), dorsal attention (44.49%), and ventral attention (67.97%) canonical networks. The network topography demonstrated high consistency across different seed radii (1 mm and 7 mm), confirming robustness.

**Conclusion:**

By integrating heterogeneous ReHo findings via FCNM, this study delineates robust PD-associated dysfunctional networks involving key sensory, motor, and attentional systems. This network-centric view offers a unifying perspective on PD pathophysiology, highlighting large-scale systems disruption and potentially reconciling previous localization inconsistencies. This approach underscores the value of network neuroscience for understanding PD mechanisms.

## Introduction

Parkinson’s disease (PD), the most common neurodegenerative movement disorder, affects approximately 1% of individuals over 60 years old (Ascherio and Schwarzschild, 2016; Tysnes and Storstein, 2017). While characterized primarily by motor deficits, PD also encompasses a significant burden of non-motor symptoms, including cognitive impairment, hallucinations, attention deficits, and depression (Marinus et al., 2018; Sveinbjornsdottir, 2016). The presence of these diverse symptoms suggests that PD pathology extends beyond the classically affected dopaminergic neurons of the substantia nigra and striatum, implicating dysfunction across a wider array of brain regions (Dayan et al., 2018; Huang C. C. et al., 2024; Lin et al., 2024; Wen et al., 2022).

To investigate the pathophysiological mechanisms underlying PD, researchers frequently utilize resting-state functional MRI (rs-fMRI) (Tessitore et al., 2019) to non-invasively assess intrinsic neural activity and functional connectivity (FC) alterations, as reflected by the blood-oxygen-level-dependent (BOLD) signal, without requiring active task participation (Allen et al., 2014). A key rs-fMRI metric employed for this purpose is regional homogeneity (ReHo), which measures the synchronicity of neural activity time courses within local brain areas. Because ReHo is calculated across the whole brain in a data-driven manner, without needing pre-defined regions of interest, it serves as a valuable tool for investigating patterns of local neural activity in both healthy individuals and patients with neurological disorders (Song et al., 2011; Zang et al., 2004; Zuo et al., 2013).

A substantial body of research has employed ReHo analysis in PD. These studies have reported significant alterations compared to healthy controls (HC), such as decreased ReHo in sensorimotor cortices and increased ReHo in parietal, occipital, and prefrontal regions, often interpreted in relation to motor deficits, sensory abnormalities, or potential compensatory neural processes (Choe et al., 2013; Li et al., 2017). However, considerable heterogeneity persists across these ReHo findings, making it challenging to establish a definitive map of consistently affected regions solely based on individual studies. This variability is frequently attributed to differences in patient demographics, clinical profiles, sample sizes, imaging acquisition parameters, and data analysis strategies. In attempts to synthesize these divergent ReHo results, coordinate-based meta-analyses (CBMA) have identified recurring patterns, including abnormal ReHo in the bilateral inferior parietal lobules, medial prefrontal cortex, superior frontal gyrus, putamen, precentral gyrus, and thalamus (Gu et al., 2022; Pan et al., 2017; Tahmasian et al., 2017; Wang et al., 2018). Despite these meta-analytic efforts, variability persists, and ongoing research continues to generate diverse findings regarding local ReHo changes in PD (Huang Z. et al., 2024; Jiang et al., 2023; Lan et al., 2023; Li K. et al., 2023; Wang et al., 2024; Wang et al., 2023), necessitating further investigation and potentially alternative explanatory frameworks.

An emerging perspective suggests that this apparent heterogeneity in focal brain abnormalities may reflect disruptions within interconnected large-scale brain networks (Fox, 2018). This network-based view posits that disease processes can manifest at different locations (nodes) within the same functionally connected system (Darby et al., 2019). Functional Connectivity Network Mapping (FCNM), a technique that integrates coordinates of structural or functional abnormalities with normative human brain connectome data, provides a powerful framework for testing this hypothesis by mapping disparate lesion or abnormality locations onto underlying brain networks (Darby et al., 2019; Peng et al., 2022). Growing evidence supports the utility of network-based approaches for understanding various neurological and psychiatric disorders (Schaper et al., 2023; Stubbs et al., 2023; Younger et al., 2023). However, despite its potential to reconcile heterogeneous findings, FCNM has been relatively underutilized in the context of ReHo alterations in PD.

Therefore, the present study aimed to apply FCNM to synthesize published findings on ReHo alterations in PD patients compared to HC. By integrating coordinate data from previous ReHo studies into a connectome framework, we seek to identify potential common functional networks underlying these alterations. This approach will allow us to investigate whether the heterogeneous regional ReHo changes reported across different studies converge onto a specific, functionally connected brain network associated with PD.

## Materials and methods

### Data sources, study selection, and quality assessment

**Figure 1:**
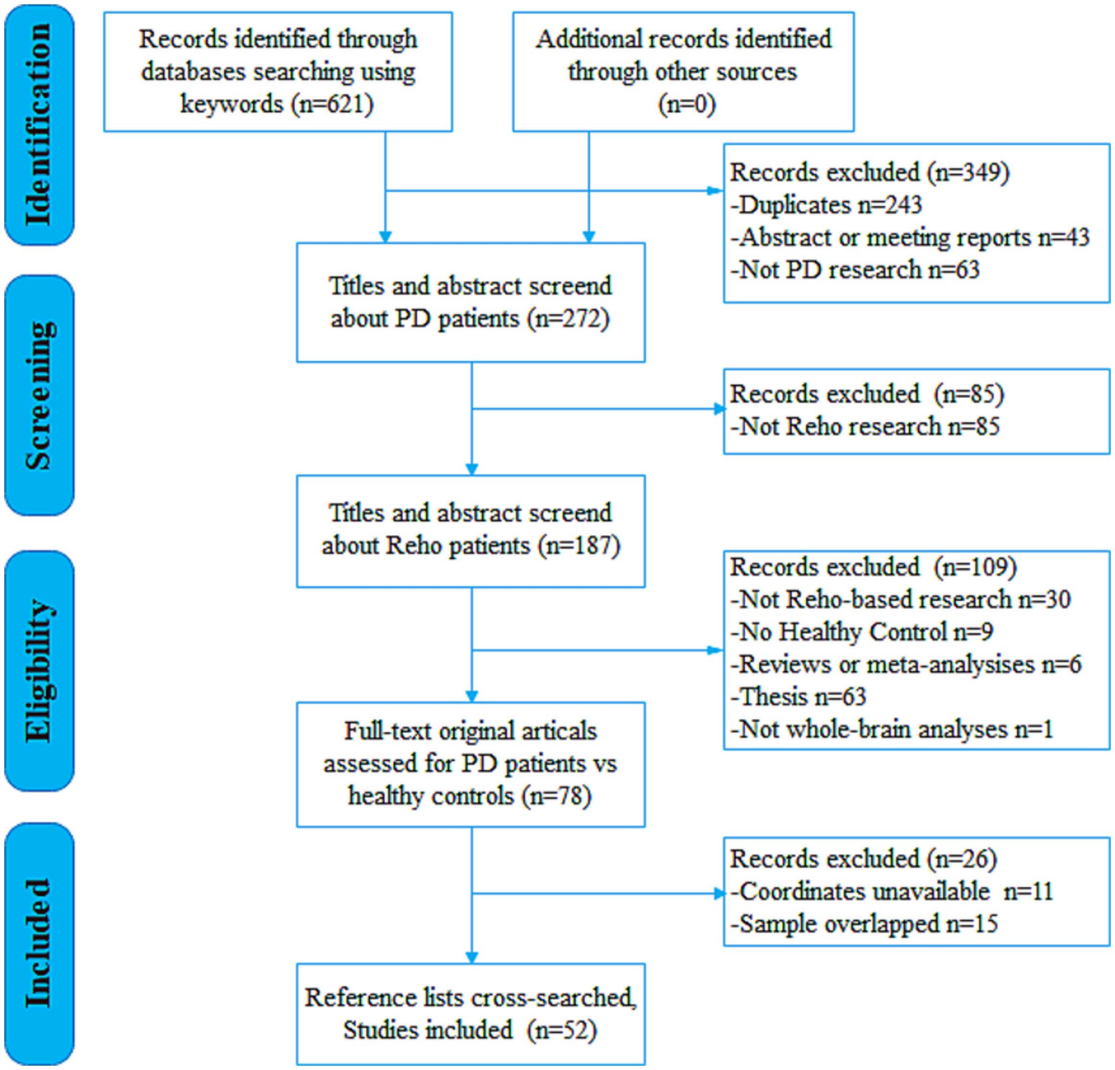
Flow diagram for the identification and exclusion of studies. PD, Parkinson’s disease; ReHo, regional homogeneity.

In accordance with Preferred Reporting Items for Systematic Reviews and Meta-Analyses (PRISMA) guidelines, a comprehensive systematic search was conducted across PubMed, Embase, Web of Science, China National Knowledge Infrastructure (CNKI), and the Chinese Wanfang Database for studies published up to April 1, 2025, using the keywords: (Parkinson^*^ OR Parkinson) AND (“regional homogeneity” OR ReHo OR “local connectivity”). To ensure thorough inclusion of relevant studies, the reference lists of selected studies and pertinent review articles were also examined. The study selection process is illustrated in the flow diagram (Figure 1).

The inclusion criteria for this study were as follows: (1) participants were diagnosed with idiopathic PD according to established clinical criteria; (2) the study involved a ReHo analysis comparing patients with idiopathic PD to HC subjects; (3) whole-brain ReHo analysis that reported three-dimensional coordinates in either the Talairach or Montreal Neurological Institute (MNI) space; (4) results achieved statistical significance, either corrected for multiple comparisons or uncorrected but employing spatial extent thresholds; and (5) the study was an original research article published in a peer-reviewed English- or Chinese-language journal. In studies that reported both on-state and off-state results, only the off-state data were included. For longitudinal studies, only data from the baseline assessment were used. If patient datasets appeared across multiple articles, only the dataset with the largest sample size and the most comprehensive reported details was chosen to avoid data duplication. Excluded items included review papers, letters, comments, and abstracts. The literature search, assessment and selection of studies, and data extraction were conducted independently by two investigators. Any discrepancies were settled by discussion involving a third investigator to reach a conclusive decision.

### Rs-fMRI data acquisition and preprocessing

For the subsequent network mapping analysis, we utilized the Human Connectome Project (HCP; http://www.humanconnectome.org/) dataset. Detailed inclusion and exclusion criteria for the HCP dataset can be found elsewhere (Marcus et al., 2013). To minimize potential confounding effects related to neurodevelopment and neurodegenerative changes, only individuals aged 18 to 40 years were included in in the present analysis. Consequently, we ultimately selected resting-state fMRI scans from 1,093 healthy participants (499 males, mean age ± SD = 28.78 ± 3.69 years).

The HCP data was acquired using a 3 T Siemens Trio scanner with a gradient echo-planar imaging (GRE-EPI) sequence for fMRI. The imaging parameters were as follows: repetition time (TR) of 720 ms, echo time (TE) of 33.1 ms, field of view (FOV) of 208 mm × 180 mm, flip angle (FA) of 52°, matrix size of 104 × 90, slice thickness/gap of 2 mm/0 mm, 72 slices, and 1,210 time points.

Resting-state fMRI data were preprocessed utilizing the Statistical Parametric Mapping (SPM12; https://www.fil.ion.ucl.ac.uk/spm/) and Data Processing & Analysis for Brain Imaging (DPABI; https://rfmri.org/DPABI) (Yan et al., 2016). The initial 10 volumes from each run were discarded to allow for MR signal stabilization and participant acclimation. The remaining volumes underwent correction for slice timing differences. Subsequently, realignment was performed to correct for inter-volume head motion. Head motion parameters were estimated (translations in x, y, z directions and rotations pitch, roll, yaw). All included participants exhibited head motion within acceptable limits (maximum translation < 2 mm and maximum rotation < 2°). Framewise displacement (FD), reflecting volume-to-volume head position changes, was also calculated. Several nuisance covariates—including linear trends, the 24 head motion parameters derived from the Friston model, volumes flagged with FD > 0.5 mm (“scrubbing”), mean global signal, mean white matter signal, and mean cerebrospinal fluid signal—were regressed out using a general linear model. Global signal regression (GSR) was included as it has been shown to enhance the specificity of functional connectivity patterns and mitigate widespread motion artifacts, although its use remains debated. The resulting datasets were then bandpass filtered (0.01–0.1 Hz). For spatial normalization, individual T1-weighted structural images were first co-registered to the mean functional image. These aligned structural images were then segmented into gray matter, white matter, and CSF probability maps and normalized to MNI standard space using the high-dimensional nonlinear warping algorithm, Diffeomorphic Anatomical Registration via Exponentiated Lie algebra (DARTEL). Each filtered functional volume was subsequently spatially normalized to MNI space using the deformation parameters derived from the structural normalization and resampled into 3-mm isotropic voxels. Finally, spatial smoothing was applied using a Gaussian kernel of 6 × 6 × 6 mm^3^ full width at half maximum (FWHM).

### Functional connectivity network mapping

We employed the FCNM approach to construct a PD-associated dysfunctional network based on the extracted coordinates of significant ReHo differences between PD and HC participants identified in the systematic review. First, spherical regions of interest (ROIs) with a radius of 4 mm were created centered at the peak coordinates reported for each significant between-group contrast (PD > HC or PD < HC). These spheres were then combined to form a contrast-specific seed mask (termed the “contrast seed”). Second, using the preprocessed normative resting-state fMRI data from the 1,093 HCP participants, functional connectivity (FC) maps were generated for each HCP participant. This involved computing the Pearson’s correlation coefficient between the mean time series extracted from the contrast seed (representing the location of PD-related ReHo abnormality) and the time series of all other brain voxels. These correlation coefficients were transformed using Fisher’s r-to-z transformation to improve normality. Third, the 1,093 individual-level z-transformed FC maps were entered into a voxel-wise one-sample *t*-test at the group level to identify brain regions demonstrating consistent functional connectivity with the contrast seed across the healthy cohort. We focused only on positive FC, given that the biological interpretation of negative FC remains a subject of ongoing research (Murphy et al., 2009; Murphy and Fox, 2017). Fourth, the resulting group-level t-map underwent thresholding using a significance level of *p* < 0.05, with correction for multiple comparisons applied using the false discovery rate (FDR) method. Finally, the thresholded, binarized maps derived from each individual ReHo contrast included in the meta-analysis were overlaid. This generated a network probability map, representing the frequency with which voxels appeared in the significant connectivity networks across different contrasts. This probability map was then thresholded at 50% (i.e., retaining voxels present in at least half of the individual contrast-derived networks) to produce the final integrated ReHo-based PD dysfunctional network.

### Association with canonical brain networks

To facilitate functional interpretation, we examined the spatial overlap between the derived PD dysfunctional network and eight well-established canonical brain networks. Following Yeo et al., the cortical networks comprised the visual, somatomotor, dorsal attention, ventral attention, limbic, frontoparietal, and default mode networks (Yeo et al., 2011). The subcortical network, encompassing the amygdala, hippocampus, basal ganglia, and thalamus, was defined using the Human Brainnetome Atlas.^1^ To quantify the spatial relationship, we calculated the proportion of overlapping voxels between the PD dysfunctional network and each canonical network, relative to the total number of voxels in the respective canonical network. If the overlap proportion reached 20% or greater, the PD dysfunctional network was considered to significantly involve the corresponding canonical network.

## Results

### Included studies and sample characteristics

Following the predefined search strategy and selection criteria, a total of 621 potentially relevant documents were screened. Ultimately, 52 studies reporting on ReHo alterations in PD, comprising 72 independent datasets (contrasts), were included in the analysis. These datasets reported ReHo differences between a pooled sample of 2,052 PD patients (1,133 males, 919 females; mean age = 60.88 ± 5.30 years; mean Hoehn & Yahr [H&Y] stage = 2.06 ± 0.67; mean disease duration = 4.77 ± 3.85 years) and 1,401 HC participants (656 males, 745 females, mean age = 60.29 ± 6.73 years). Detailed sample and imaging characteristics of the included studies are summarized in Supplementary Table 1.

### Dysfunctional networks in PD

**Figure 2:**
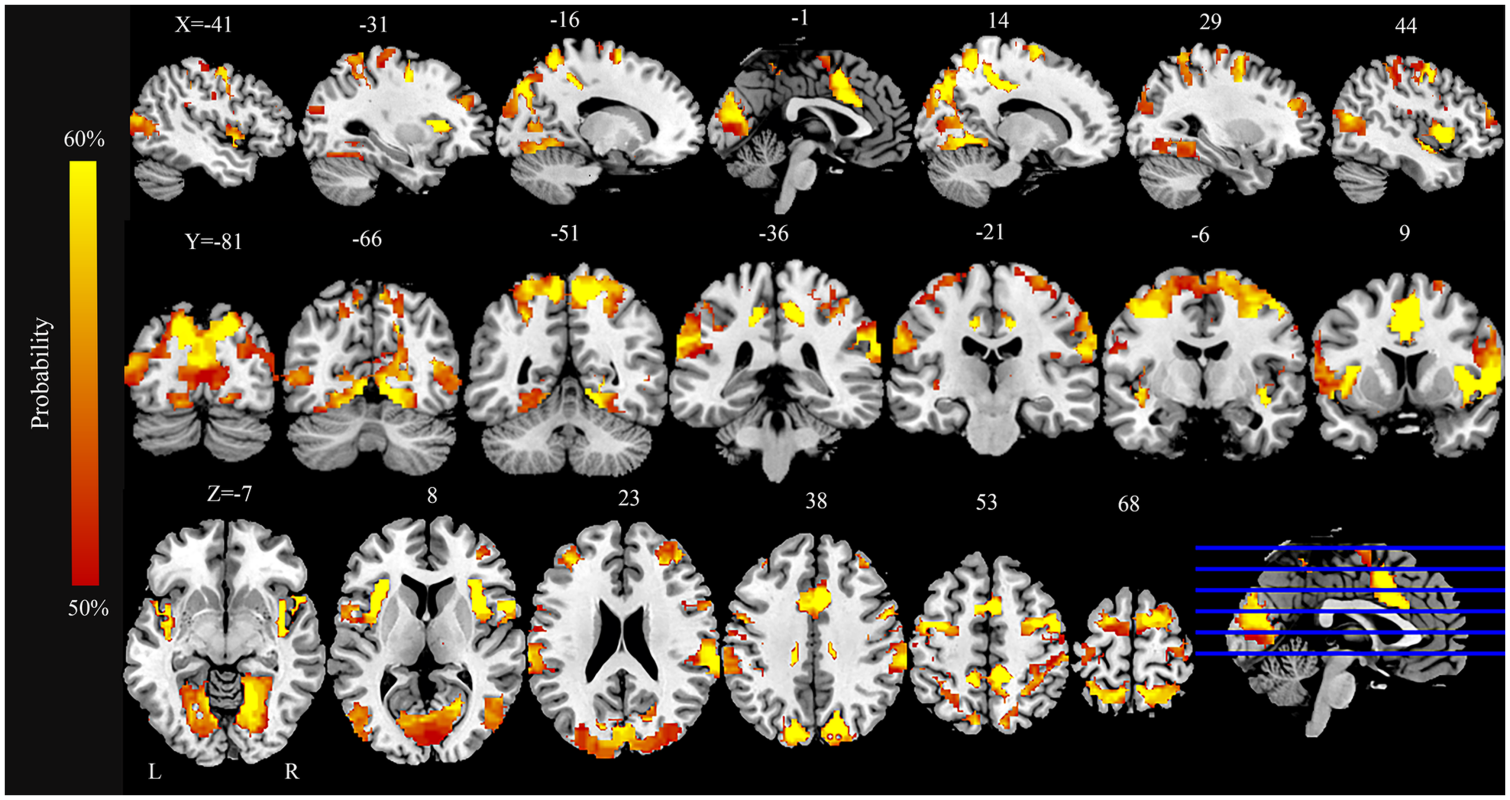
PD brain damage networks and involved brain regions. PD, Parkinson’s disease.

The FCNM analysis integrating coordinates of ReHo alterations revealed a PD-associated dysfunctional network comprising a broadly distributed set of brain regions (Figure 2). Key nodes included extensive areas within the bilateral occipital cortex (lingual gyrus, calcarine cortex, cuneus, superior occipital gyrus, and middle occipital gyrus), somatomotor cortex (precentral gyrus, postcentral gyrus, and supplementary motor area), parietal cortex (superior parietal gyrus, precuneus, and inferior parietal lobule), and the insula.

**Figure 3:**
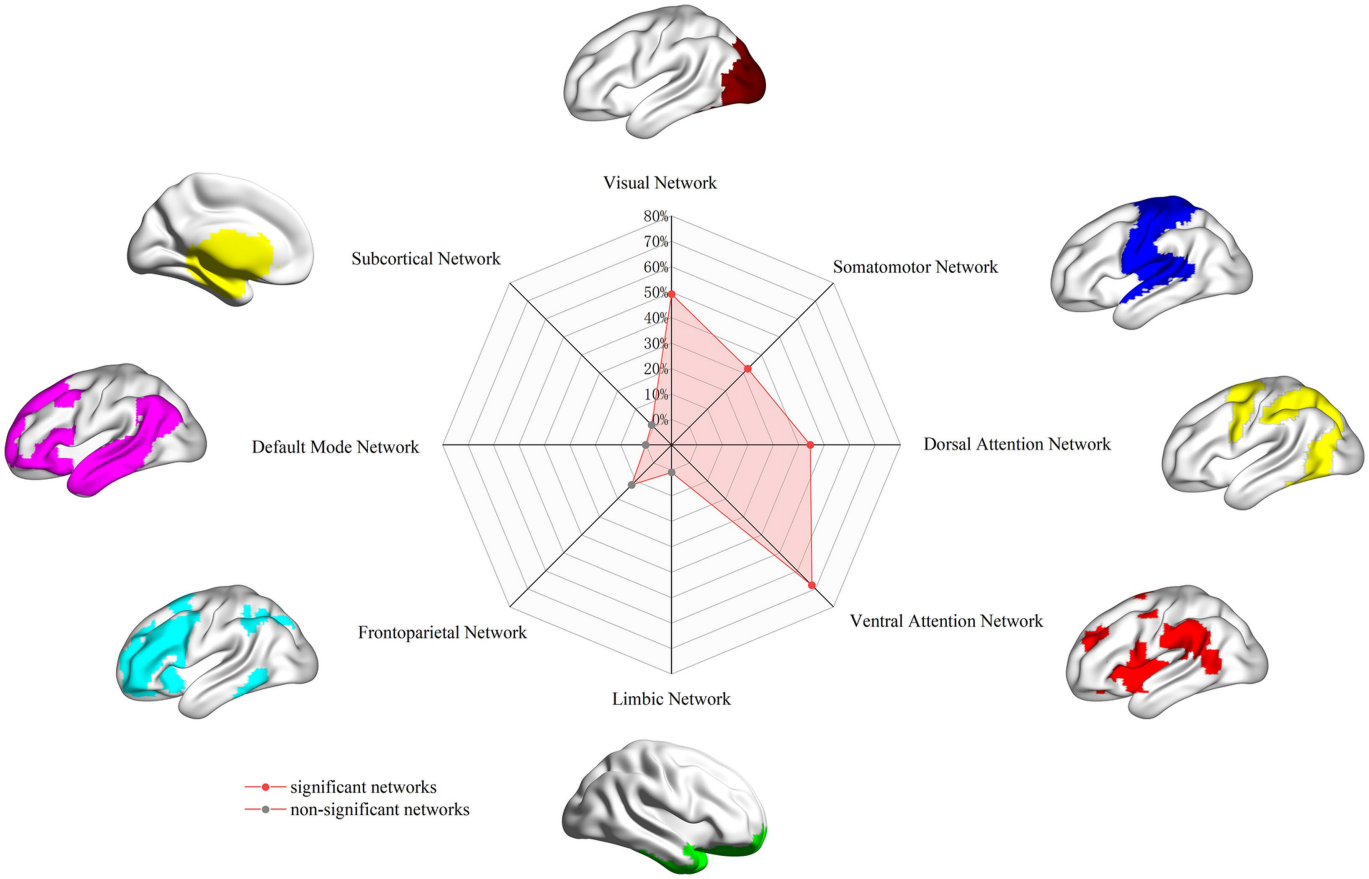
PD brain damage network in association with canonical brain networks. PD, Parkinson’s disease.

Regarding overlap with canonical networks (Figure 3), the PD dysfunctional network primarily involved the visual (overlap proportion: 49.24%), somatomotor (32.35%), dorsal attention (44.49%), and ventral attention (67.97%) networks, all exceeding the predefined 20% threshold. To evaluate the robustness of the FCNM procedure to the choice of seed radius, analyses were repeated defining seed spheres with radii of 1 mm and 7 mm. The resulting PD dysfunctional networks closely resembled the network generated using the primary 4-mm sphere radius. Specifically, with a 1-mm radius, the significantly involved canonical networks included the visual (30.45%), dorsal attention (27.16%), and ventral attention (57.95%) networks (somatomotor network overlap was 13.9%, below threshold). With a 7-mm radius, the significantly involved networks included the visual (45.72%), somatomotor (34.65%), dorsal attention (37.52%), and ventral attention (69.77%) networks, demonstrating high consistency across radii.

## Discussion

To the best of our knowledge, this study represents the first application of FCNM, integrating coordinate-based data of ReHo alterations with large-scale normative human connectome data, to delineate the PD-associated dysfunctional networks. By synthesizing 72 contrasts from 52 studies, encompassing a substantial cohort of 2,052 PD patients and 1,401 HC, our FCNM analysis identified consistent dysfunctional networks associated with PD. The results revealed that this PD-related networks primarily involve nodes within the visual, somatomotor, dorsal attention, and ventral attention canonical systems. The robustness of this network topography was confirmed through validation analyses using different seed radii (1 mm and 7 mm), which yielded highly comparable results to the primary analysis (4 mm radius). This network-level perspective offers a potential framework for reconciling previously heterogeneous regional neuroimaging findings and provides a systems-level vantage point for understanding the neurobiological underpinnings of PD.

Our analysis identified significant involvement of the visual network in PD, including nodes located within cortical regions such as the bilateral lingual gyrus, calcarine cortex, cuneus, superior occipital gyrus, and middle occipital gyrus. The visual network is integral to processing external visual stimuli, encompassing functions like visual perception, object recognition, spatial localization, and the regulation of visual attention—domains known to be affected in PD (Xing et al., 2024). Clinically, visual network impairment in PD manifests diversely, including reduced visual acuity and contrast sensitivity, deficits in visual scene processing, visuospatial cognition, color vision, and the occurrence of visual hallucinations (Archibald et al., 2013; Armstrong, 2011; Caproni et al., 2014; Manganelli et al., 2009; Norton et al., 2016; Sun et al., 2014). Indeed, visual problems are highly prevalent, affecting up to 70% of PD patients (Urwyler et al., 2014), and can emerge even in the prodromal phase (Armstrong, 2015; Mahlknecht et al., 2015). The implication of the visual network identified through our synthesis of ReHo data aligns with previous multimodal neuroimaging evidence. For instance, studies have reported altered functional organization (segregation/integration) within dorsal and ventral visual streams (Li T. et al., 2023), reduced metabolic activity (FDG-PET) (Zang et al., 2023), and abnormal neurovascular coupling (Li T. et al., 2023) within this network in PD. Furthermore, the specific regions highlighted in our network map, such as the lingual gyrus, calcarine cortex, cuneus, and occipital gyri, have been repeatedly implicated in prior functional connectivity (Kawabata et al., 2018) and meta-analytic studies focusing on local activity alterations in PD (Gu et al., 2022; Pan et al., 2017; Wang et al., 2018; Wang et al., 2023), supporting the notion that widespread visual system dysfunction is a core feature captured by aggregating ReHo findings.

This study also demonstrated significant involvement of the somatomotor network (SMN) in the PD dysfunctional network, including key nodes in the precentral gyrus, postcentral gyrus, and supplementary motor area (SMA). The SMN plays a critical role in coordinating and integrating sensorimotor information (Marquez et al., 2023), and its functional architecture reflects the brain’s regulation of motor control (Wang et al., 2022; Yeo et al., 2011). In PD, dysfunction within the SMN is intimately linked to cardinal motor symptoms, particularly impacting motor planning and gait initiation (Ragothaman et al., 2022). Consistent with this, SMN functional connectivity has been shown to predict clinical motor scores (Wang et al., 2022), and alterations in SMN metabolic activity correlate with motor severity (Zang et al., 2023). Moreover, therapeutic interventions like levodopa have been shown to modulate SMN synchrony in correlation with motor improvements (Zhou et al., 2021). The specific SMN regions identified in our FCNM analysis (precentral/postcentral gyri, SMA) correspond well with established patterns of PD pathology. Abnormalities in the precentral and postcentral gyri are frequently reported (Li et al., 2021; Wang et al., 2024; Zeng et al., 2017) and functional and structural alterations within the SMA are strongly associated with characteristic PD deficits such as impaired motor sequencing, timing, and gait (Marquez et al., 2023). Notably, the SMA is a target for therapeutic interventions, with repetitive transcranial magnetic stimulation (rTMS) applied to this area showing moderate efficacy in improving motor symptoms in randomized controlled trials (Hamada et al., 2008; Ramos et al., 2013). This convergence of evidence underscores the clinical relevance of the SMN components highlighted by our FCNM integration of ReHo data.

Additionally, our analysis revealed widespread involvement of attention networks, including both the dorsal attention network (DAN) and the ventral attention network (VAN), encompassing regions such as the superior parietal lobule, precuneus, inferior parietal lobule, and insula. These networks are fundamental components of cortical organization, interacting with sensory systems to regulate attention and facilitate information processing (Yeo et al., 2011). Evidence suggests disrupted attentional network function in PD; for example, increased network dispersion in both VAN and DAN has been observed (Zang et al., 2023). The VAN plays a critical role in directing attention toward unexpected or salient stimuli, operating as a bottom-up, stimulus-driven attentional process, while the DAN is responsible for top-down, goal-directed stimulus selection (Rossi et al., 2014; Vossel et al., 2014). PD patients exhibit impairments related to both systems: difficulties in recognizing salient targets (implicating DAN/top-down control) and altered orienting to novel stimuli (implicating VAN/bottom-up control), particularly in fatigued patients (Pauletti et al., 2019). Research suggests FOG may involve VAN overreliance on external cues, leading to executive deficits (Bayot et al., 2022), and alterations in DAN network efficiency (Maidan et al., 2019). Beyond overt attention and FOG, DAN alterations have been implicated in broader cognitive processing and impulse control disorders in PD (Arslan et al., 2020; Baggio et al., 2015; Chung et al., 2019; Zhu et al., 2021). Furthermore, disrupted processing within and between attention networks is hypothesized to contribute to visual illusions and hallucinations. For instance, impaired communication between the VAN (detecting saliency) and DAN (directing focus) might lead to misidentification of stimuli, allowing internally generated percepts to emerge (Gobel et al., 2021; Shine et al., 2014a). Importantly, the cortical regions associated with the attention networks in our FCNM results (superior/inferior parietal lobules, precuneus, insula) align well with previous meta-analyses and studies reporting altered activity, connectivity, or synchrony in these areas in PD (Gu et al., 2022), demonstrating that the attention network nodes identified via our synthesis of ReHo data correspond to regions consistently implicated in PD attentional and related cognitive dysfunction.

Beyond characterizing alterations within individual canonical networks, impaired interactions between these networks likely contribute significantly to the pathophysiology of PD. For instance, effective gait control requires coordinated activity between the visual network (for spatial guidance) and the SMN (for motor execution) (Takakusaki, 2013). Reduced functional connectivity between these two systems in PD patients is thought to impair sensorimotor integration, contributing to postural instability and gait difficulties (Gratton et al., 2019; Shi et al., 2023; Xing et al., 2024). Highlighting this interaction, a predictive model for PD motor dysfunction identified Visual-SMN network coupling as a key factor (Wang et al., 2022). Similarly, the occurrence of visual hallucinations likely involves multi-network dysregulation. Models propose that reduced DAN activity, hyperactivation of the VAN, and impaired DAN-VAN connectivity create a vulnerability (Diez-Cirarda et al., 2023; Nieto-Escamez et al., 2023; Pagonabarraga et al., 2024; Shine et al., 2014a,b), where ambiguous visual input, coupled with dysfunctional VAN processing, allows intrusive memory-based imagery to manifest as hallucinations (Zhang et al., 2024). This dysregulation is a key factor in the development of hallucinations in PD psychosis (Pagonabarraga et al., 2024). Therefore, while our FCNM approach primarily identifies the topography of the dysfunctional network based on ReHo alterations, these findings gain further significance when considering the critical functional interactions between the implicated visual, somatomotor, and attention systems in producing the complex symptom profile of PD.

However, in the present study, the FCNM analysis based on pooled ReHo coordinates did not highlight the default mode network (DMN) as a significantly altered network in PD, unlike in many studies focusing on specific subgroups. We speculate that this is not simply attributable to the relatively short disease duration, but rather likely reflects the significant heterogeneity of DMN functional alterations within the PD patient population. Extensive literature indicates substantial variability in DMN functional connectivity patterns among PD patients, which is closely related to the patients’ cognitive status (even within the non-demented range, such as distinguishing between cognitively normal and mild cognitive impairment) (Hou et al., 2020; Zarifkar et al., 2021), specific clinical phenotypes (e.g., the presence of visual hallucinations) (Shine et al., 2015; Yao et al., 2014), and the functional specificity of DMN internal subsystems (Zarifkar et al., 2021). Furthermore, DMN dysfunction often manifests as altered interaction patterns with other large-scale brain networks (such as the salience and executive control networks) (Putcha et al., 2016; Tessitore et al., 2012). ReHo primarily measures the synchrony of local neural activity; pooling its coordinates across a clinically diverse group likely averages out or obscures these heterogeneous DMN alteration patterns, which can be in opposite directions, involve specific subnetworks, or pertain to inter-network interactions. Therefore, our findings might indirectly underscore the complexity of DMN dysfunction within the PD spectrum, suggesting the need for more refined subgroup analyses or the use of different methodologies (e.g., direct functional connectivity analysis) to capture its changes.

Despite the strengths of our approach, several limitations must be acknowledged. First, our study was constrained to studies reporting three-dimensional coordinates for ReHo differences, potentially excluding relevant studies that used different analytical methods (e.g., region-of-interest analyses) or did not report coordinates, which could introduce selection bias. Second, the normative connectome used for FCNM was derived from the HCP dataset, comprising young healthy adults (18–40 years). The demographic profile of this normative sample differs significantly from the typically older PD patient population included in the included studies. Using an age-matched normative connectome, if available, might provide more precise network mapping in future studies. Third, while FCNM provides a valuable framework for integrating localized findings, it is a relatively recent technique whose application to diverse neurological conditions warrants further validation in independent, large-scale cohorts to confirm robustness and generalizability. Fourth, while FCNM helps synthesize heterogeneous findings, inherent variability within the included studies (e.g., clinical heterogeneity of patients, differences in imaging protocols and preprocessing pipelines beyond ReHo calculation) remains a factor that influences the input coordinates and, consequently, the derived network map. Finally, we acknowledge that not all included studies reported results corrected for multiple comparisons. Whenever both corrected and uncorrected results were available, we prioritized using statistically corrected findings. However, in some studies, only uncorrected results were provided, which we included to ensure comprehensiveness. This may have introduced some variability and should be considered when interpreting the findings.

## Conclusion

In conclusion, this study successfully employed FCNM by integrating coordinate-based results from numerous rs-fMRI studies investigating ReHo alterations in PD. Our analysis revealed consistent and robust PD-associated dysfunctional networks, primarily implicating the visual, somatomotor, dorsal attention, and ventral attention networks. This network-centric approach offers a potentially unifying framework that may help reconcile previously heterogeneous findings focused on isolated brain regions. By mapping disparate ReHo alterations onto interconnected functional systems, our findings provide novel insights into the systems-level pathophysiology underlying PD, linking widespread changes in local neural synchrony to specific large-scale networks known to be involved in the visual, motor, and attentional deficits characteristic of the disease. While acknowledging limitations related to coordinate-based synthesis and the normative dataset employed, this work underscores the value of network neuroscience perspectives in understanding complex neurodegenerative disorders. It highlights that PD pathology manifests across interconnected brain systems and paves the way for future investigations utilizing network-based analyses to further explore disease mechanisms, track progression, and potentially identify novel therapeutic targets within these affected circuits.

## Data Availability

The original contributions presented in the study are included in the article/Supplementary material, further inquiries can be directed to the corresponding authors.

## Footnotes

^1^ https://atlas.brainnetome.org/
