# Supplementary material for: Network localization of regional homogeneity alterations in Parkinson’s disease
Source: Front Aging Neurosci. 2025 May 19;17:1607691. doi: 10.3389/fnagi.2025.1607691 (PMC12127408; doi:10.3389/fnagi.2025.1607691)
Supplement: Supplementary file 1 [file Table_1.DOCX]

**Supplementary Table 1**

Clinical characteristics and scanning modalities of ReHo studies of PD versus HC

| **Study** | **Language** | **Number/female** | |  | **Age(years)** | | **UPDRS** | **H&Y stage** | **Disease duration** | **Medication State** | **Scanner** | **Softer ware** | **FWHM** | **Threshold** |
| --- | --- | --- | --- | --- | --- | --- | --- | --- | --- | --- | --- | --- | --- | --- |
|  |  | **PD** | **HC** |  | **PD** | **HC** |  |  |  |  |  |  |  |  |
| (Wu et al., 2009) | English | 22/6 | 22/6 |  | 59.5 ± 8.1 | 59.7 | 25.6 ± 8.1 | 1.7 ± 0.5 | 4.1 ± 1.8 | ON/OFF | 1.5T | AFNI | 4mm | p < 0.05 corrected |
| (Liu et al., 2011) | Chinese | 16/8 | 12/6 |  | 59.1 ± 8.7 | 60.1±8.2 | 28.2±4.8 | NA | 4.9 (2-11) | OFF | 3.0T | SPM 5 | 4mm | p < 0.05 uncorrected |
| (Xian Liu, 2011) | English | 9/5 | 8/3 |  | 64.78±7.58 | 65.13±8.15 | 30.28±3.36 | 2.44±0.53 | 4.22±2.49 | OFF | 1.5T | ReHo fMRI | 4mm | p < 0.05 corrected |
| (Yeo et al., 2012) | English | 12/6 | 12/6 |  | 53.5±10.9 | 55.9 ± 9.8 | 7.8 ±3.9 | 1.5±0.6 | 2.67±2.3 | OFF | 3.0T | SPM 5 | 9mm | p < 0.05 corrected |
| (Liu et al., 2012) | Chinese | 9/3 | 8/NA |  | 64.78±7.58 | NA | 30.5±2.98 | 2.67±0.50 | NA | OFF | 1.5T | REST | NA | p<0.001 uncorrected |
| (Yang et al., 2013) | English | 17/7 | 17/7 |  | 60.43±9.65 | 60.73±8.57 | NA | NA | NA | OFF | 1.5T | AFNI, REST | 4mm | p < 0.05 corrected |
| (Choe et al., 2013) | English | 22/12 | 25/15 |  | 58.3±2.4 | 58.3±1.7 | 10.4±1.2 | 1.6±0.2 | 3.2±0.4 | OFF | 3.0T | DPARSF | 9mm | p < 0.05 corrected |
| (Wang et al., 2014) | Chinese | 30/10 | 30/12 |  | 63±2 | 61±3 | NA | NA | 5±2 | NA | 3.0T | AFNI, SPM 5, REST | NA | p < 0.05 |
| (Sheng et al., 2014) | English | ^a^21/8  ^b^20/7 | 25/9 |  | ^a^55.96*±*7.4  ^b^57.36*±*6.1 | 56.76*±*5.3 | ^a^39.46 *±* 10.8  ^b^43.8 *±* 68.2 | NA | ^a^3.46±1.7  ^b^4.0±2.4 | OFF | 3.0T | SPM 8, REST, DPARSFA | 4mm | p < 0.05 corrected |
| (Borroni et al., 2015) | English | 11/1 | 10/7 |  | 66.3±3.8 | 62.2±8.0 | 10.7 ± 5.4 | NA | 7.8 ± 3.1 | NA | 1.5T | SPM 8, REST, DPARSFA | 8mm | p<0.05  corrected |
| (Zhang et al., 2015) | English | ^c^20/10  ^d^27/11 | 26/15 |  | ^c^54.55±12.58  ^d^63.38±9.46 | 59.31±7.15 | ^c^19.35 ±9.35  ^d^19.88 ± 6.70 | ^c^1.88 ± 0.70  ^d^2.21 ± 0.67 | ^c^5.0±3.21  ^d^4.17±4.07 | OFF | 3.0T | SPM 8, REST | 4mm | p < 0.05 corrected |
| (Feng et al., 2015) | Chinese | 29/22 | 20/15 |  | 59.79±10.29 | 60.10±13.92 | NA | NA | NA | OFF | 3.0T | SPM 8, REST, DPARSF | 4mm | p < 0.05 corrected |
| (Wen et al., 2016) | English | 31/12 | 31/16 |  | 60.1 ± 8.17 | 59.6 ± 7.65 | NA | NA | NA | OFF | 3.0T | REST, DPARSF | 4mm | p < 0.05 corrected |
| (Li et al., 2016) | English | 23/12 | 20/9 |  | 63.0 ± 7.1 | 65.3 ± 7.0 | 38 ±18.6 | 2.2 ± 0.9 | 7 ± 3.3 | OFF | 3.0T | SPM 5, DPARSF | 8mm | p<0.001 |
| (Harrington et al., 2017) | English | 31/9 | 30/19 |  | 67.4 ± 7.5 | 68.6 ± 7.2 | NA | NA | 5.4±3.8 | OFF | 3.0T | AFNI | 4mm | p < 0.05 corrected |
| (Zhong et al., 2018) | Chinese | 32/12 | 32/14 |  | 63.2 ± 5.5 | 63.7 ± 6.24 | NA | NA | NA | NA | 3.0T | DPARSF | NA | p < 0.05 corrected |
| (Sun et al., 2018) | Chinese | ^a^42/20  ^b^20/11 | 47/24 |  | ^a^57.86 ± 6.71  ^b^57.45±7.10 | 57.57 ± 5.61 | ^a^25.20 ± 12.87  ^b^27.25 ± 12.59 | ^a^1.76 ± 0.66  ^b^1.38 ± 0.58 | ^a^6.12±3.39  ^b^5.40±2.84 | NA | 3.0T | DPARSF  SPM 8 | 4mm | p < 0.05 |
| (Ma et al., 2018) | Chinese | 18/10 | 20/11 |  | 70.94 ± 4.95 | 71.00 ± 6.45 | 20.31±4.47 | 2(1.5, 2.5) | 1.75 (1, 3) | OFF | 3.0T | REST | 4mm | p < 0.01 corrected |
| (Wang et al., 2019) | Chinese | ^c^43/12  ^d^34/10 | 29/13 |  | ^c^63.5 ± 8.9  ^d^66.1 ± 3.1 | 65.1 ± 4.3 | ^c^21.6 ± 10.7  ^d^24.8 ± 9.0 | NA | ^c^3.0(1.5, 5.0)  ^d^3.2(2.0, 5.2) | OFF | 3.0T | DPARSF, REST | NA | p < 0.05 |
| (Chen et al., 2019) | English | ^e^32/14  ^f^24/6 | 23/12 |  | ^e^65.50±7.73 ^f^63.04±5.65 | 61.43±8.46 | ^e^28.50±16.12  ^f^25.79±13.36 | ^e^1.91±0.50  ^f^2.04±0.62 | ^e^5.44±3.88 ^f^5.17±3.91 | OFF | 3.0T | DPABI,  SPM 12 | NA | p < 0.05 corrected |
| (Li et al., 2019) | English | ^g^14/9  ^h^20/8 | 19/12 |  | ^g^62.86±2.25  ^h^64.60±1.29 | 62.84±1.61 | ^g^31.14±4.23  ^h^29.20±2.61 | ^g^2.11±0.20  ^h^1.92±0.11 | NA | OFF | 3.0T | DPABI | NA | p < 0.05  corrected |
| (Liu et al., 2019) | English | ^i^33/12  ^j^35/16 | 35/24 |  | ^i^68*.*91 ± 8*.*17  ^j^62*.*60±10*.*22 | 59.57±5.94 | ^i^31*.*80±18*.*35  ^j^27*.*48±13*.*36 | ^i^2*.*70±0*.*74  ^j^2*.*03±0*.*52 | ^i^5.81±3.88  ^j^3.30±3.04 | ON | 3.0T | DPABI, REST, SPM 12 | 4mm | p < 0.05 corrected |
| (Oh et al., 2020) | English | 68/46 | 70/50 |  | 69.7±7.8 | 67.8 ± 6.9 | 23.3±9.5 | NA | NA | NA | 3.0T | AFNI, SPM 8, DPARSF | 4mm | p < 0.05 corrected |
| (Li et al., 2020a) | English | 24/20 | 25/21 |  | 53.13±8.49 | 49.12 ± 11.81 | 24.00 ± 16.47 | 2.20 ± 0.87 | 3.35±3.36 | OFF | 3.0T | DPABI | 8mm | p < 0.05 corrected |
| (Li et al., 2020b) | English | ^k^25/11  ^l^25/14 | 25/13 |  | ^k^63.08±7.45  ^l^59.44 ± 9.23 | 61.76 ± 6.53 | ^k^23.64 ± 11.18  ^l^20.24 ± 12.84 | NA | ^k^4.58 ± 2.66  ^l^3.16 ± 1.93 | ON | 3.0T | SPM12, DPABI | 4mm | p < 0.05  corrected |
| (Liu et al., 2020a) | Chinese | 40/20 | 45/23 |  | 63.4±6.5 | 61.9±5.2 | NA | NA | 8.8±6.4 | NA | 3.0T | DPARSF  REST | 3mm | p < 0.05 |
| (Liu et al., 2020b) | Chinese | 23/15 | 27/13 |  | 51.68 ± 6.17 | 49.55 ± 3.70 | NA | NA | NA | NA | 1.5T | SPM 8, DPARSF | 4mm | p < 0.01  corrected |
| (Yue et al., 2020) | English | ^m^14/6  ^n^26/10 | ^o^10/5  ^p^10/7 |  | ^i^49*.*8 ± 3*.*6  ^n^60*.*8 ± 3*.*5 | ^m^49*.*7 ± 2*.*3  ^n^58*.*0 ± 4*.*1 | ^m^21.1± 8.3  ^n^19.8 ± 10.5 | ^m^1*.*71 ± 0*.*61  ^n^1*.*42 ± 0*.*58 | ^m^2.24±1.42#  ^n^1.83±1.46# | OFF | 3.0T | REST | 6mm | p<0.05  corrected |
| (Sun et al., 2020) | English | ^q^20/6  ^r^26/12 | 23/10 |  | ^q^59.85 ± 8.92  ^r^59.61 ± 9.97 | 59.47±10.79 | ^q^18.05 ± 3.03  ^r^17.08 ± 3.74 | ^q^1.90 ± 0.60  ^r^1.94 ± 0.62 | ^q^2.05±0.81  ^r^1.98±0.87 | Drug-naïve | 3.0T | SPM12, DPABI | 4mm | p < 0.05  corrected |
| (Zhu et al., 2020) | Chinese | 16/6 | 12/7 |  | 63.8±8.5 | 63.9±4.6 | 24.8±10.8 | 2.2±0.5 | 3.8±2.5 | OFF | 3.0T | DPARSF | NA | p < 0.05 uncorrected |
| (Qiu et al., 2020) | Chinese | ^a^22/9  ^b^23/7 | 25/16 |  | ^a^64.5±17.0  ^b^66.0±23.0 | 55.0±11.5 | ^a^39.0±17.3  ^b^35.0±9.0 | ^a^2.0±1.5  ^b^2.0±1.0 | ^a^3.0±4.5  ^b^3.0±4.5 | OFF | 3.0T | DPABI,  SPM8 | NA | p < 0.05 |
| (Peng et al., 2020) | Chinese | ^k^12/7  ^l^10/6 | 25/14 |  | ^k^66.66±6.34  ^l^65.50±7.46 | 63.12±4.76 | NA | ^k^2.25±0.50  ^l^2.00±0.47 | ^k^4.41±1.64  ^l^4.20±1.14 | OFF | 3.0T | SPM 8, REST | NA | p < 0.05 |
| (Li et al., 2021) | English | 75/33 | 37/20 |  | 58.95±9.55 | 58.05±8.78 | 15.3 ± 10.42 | 1.63 ± 0.60 | 1.97±1.73# | OFF | 3.0T | REST | 6mm | p<0.05 |
| (Guo et al., 2021) | English | ^k^18/8  ^l^16/9 | 20/8 |  | ^k^68.33±5.88  ^l^65.50±3.81 | 61.50 ± 6.79 | ^k^44(14, 56)  ^l^17(10, 34.5) | ^k^2.5(1.0, 3.0)  ^l^2.0(2.0, 3.0) | ^k^8.0(2.0, 6.0)  ^l^5.0(1.0, 6.5) | OFF | 3.0T | DPABI,  SPM 12 | 6mm | p < 0.05  corrected |
| (Luo et al., 2021) | English | 27/11 | 12/6 |  | 62.30 ± 8.97 | 63.92 ± 8.38 | 37.70 ± 12.04 | NA | 8.26±2.78 | OFF | 1.5T | DPABI, SPM12 | 4mm | p < 0.05 corrected |
| (Xing et al., 2021) | English | ^k^ 30/14  ^l^ 19/5 | 21/13 |  | ^k^ 61.37±11.37  ^l^ 58.74±10.93 | 60.76±5.22 | ^k^30.55(17.11–40.22)  ^l^21.34(15.24–29.56) | ^k^2.4(1.0–3.0)  ^l^1.5(1.0–2.5) | ^K^3.1(1.5–6.5)  ^l^2.5(1.0–4.7) | NA | 3.0T | DPARSF, SPM12 | 8mm | p < 0.05 corrected |
| (Cao et al., 2021) | Chinese | 58/27 | 35/22 |  | 58.2 ± 10.4 | 59.8 ± 9.9 | NA | NA | NA | OFF | 3.0T | SPM 12, DPARSFA, DPABI | 4mm | p < 0.05 corrected |
| (Wang et al., 2021) | English | 50/27 | 30/10 |  | 60.43 ± 7.47 | 56.64 ± 7.83 | 28.37 ± 1.58 | NA | ^s^1.28±0.71  ^t^1.11±0.67 | Drug-naïve | 3.0T | SPM 12, REST | 3mm | p < 0.05 corrected |
| (Zhang et al., 2022) | English | ^s^41/24  ^t^39/22 | 40/24 |  | ^s^65.54 ± 9.6  ^t^65.62 ± 10.23 | 60.97 ± 8.82 | ^s^28.45 ± 12.92  ^t^21.80 ± 12.96 | ^s^1.85 ± 0.59  ^t^1.73 ± 0.55 | ^s^3.28±2.29#  ^t^3.38±2.95# | OFF | 3.0T | SPM12, DPABI | NA | p < 0.05  corrected |
| (Zhu et al., 2022) | English | 20/7 | 20/10 |  | 67.4 ± 7.4 | 65.4 ± 4.4 | 34.3 ± 9.3 | 3.1 ± 0.9 | 3.9±2.9 | OFF | 3.0T | Resting-State fMRI V2.1 | 6mm | p < 0.05 corrected |
| (Lu et al., 2022) | Chinese | 32/22 | 33/16 |  | 64.7 (55.8-72.8) | 62.8 (59.0～67.0) | 25.8±16.2 | NA | NA | OFF | 3.0T | DPABI, SPM 12 | 6mm | p<0.05 |
| (Jiang et al., 2022) | English | ^u^23/5  ^v^27/9 | 26/12 |  | ^u^66.65 ± 7.06  ^v^63.26 ± 7.56 | 62.73 ± 6.33 | ^u^31.48 ± 11.51  ^v^29.89 ± 15.25 | ^u^2.52 ± 0.86  ^v^2.50 ± 0.89 | ^u^7.30±4.42  ^v^6.04 ± 3.46 | OFF | 3.0T | DPARSF, REST, SPM 8 | 4mm | p < 0.05 corrected |
| (Xu et al., 2022) | English | ^q^28/18  ^r^19/8 | 32/19 |  | ^q^65.1 ± 6.2  ^r^60.3 ± 11.3 | 63.2 ± 4.7 | ^q^39.0 ± 18.2  ^r^26.9 ± 13.5 | ^q^2.1 ± 0.8  ^r^1.4 ± 0.5 | ^q^5.4±4.5  ^r^3.2±3.3 | OFF | 3.0T | REST, DPABI | 6mm | p < 0.05  corrected |
| (Wang et al., 2023) | English | 38/12 | 35/19 |  | 58.82±7.78 | 58.80±5.51 | 36.47±17.04 | 2.30±1.00 | 5.18±3.46 | ON | 3.0T | REST | 6mm | p < 0.05  corrected |
| (Li et al., 2023) | English | 57/29 | 31/15 |  | 64.16 *±* 8.13 | 62.42 *±* 7.19 | 49.90 *±* 18.82 | 2.20 *±* 0.69 | 6.49±3.59 | OFF | 3.0T | REST, SPM 12, DPABI | 6mm | p < 0.05  corrected |
| (Zang et al., 2023) | Chinese | 34/21 | 25/17 |  | 62.3 ± 6.4 | 60.0±4.5 | 59.71±15.00 | 3.00±0.83 | NA | OFF | 3.0T | SPM | 8mm | p < 0.05 |
| (Jiang et al., 2023) | English | ^w^50/17  ^x^53/18 | 20/4 |  | ^w^61.65 ± 9.19  ^x^61.41 ± 10.72 | 63.73 ± 9.85 | ^w^21.58 ± 11.29  ^x^22.06 ± 11.12 | ^w^1.76 ± 0.48  ^x^1.70 ± 0.54 | NA | Drug-naïve | 3.0T | SPM12, DPARSF | 4mm | p < 0.05 corrected |
| (Lan et al., 2023) | English | ^c^21/13  ^d^28/12 | 32/15 |  | ^c^62.2±9.38  ^d^62.6±6.83 | 61.2±7.91 | ^c^53.57 ± 17.17  ^d^54.43 ± 14.11 | ^c^2.31 ± 0.46  ^d^2.86 ± 0.43 | ^c^7.24 ± 4.95  ^c^8.43 ± 4.63 | OFF | 3.0T | REST, SPM12, | NA | p < 0.05  corrected |
| (Gong et al., 2024) | Chinese | 24/6 | 23/9 |  | 69.08 ± 8.03 | 67.43±6.99 | 19.6 ± 9.1 | 2.5 (2, 3) | NA | OFF | 3.0T | SPM12, DPABI | NA | p<0.05  Bonferroni correction |
| (Huang et al., 2024) | Chinese | 29/10 | 44/23 |  | 66.07±9.17 | 66.32±4.44 | NA | NA | NA | NA | 3.0T | DPABI,  SPM 12 | 6mm | p < 0.05  corrected |
| (Wang et al., 2024) | English | 66/27 | 57/33 |  | 61.08±8.56 | 59.05±5.97 | 32.41±15.55 | NA | 4(1.5,7) | NA | 3.0T | RESTplus | 6mm | p < 0.05 corrected |
| (Dai et al., 2025) | English | ^c^29/7  ^d^30/14 | 30/16 |  | ^c^63.14±6.83  ^d^64.83±9.40 | 62.93±9.66 | ^c^17.90±6.816 ^d^23.31±11.149 | ^c^1.53±0.53  ^d^2.62±1.00 | NA | OFF | 3.0T | RESTplus | 6mm | p < 0.05 corrected |

Data are presented as mean (± standard deviation) or median (interquartile range).# Indicates values derived from original reports with disease duration in months, converted into years for consistency.

ReHo, regional homogeneity; PD: Parkinson’s disease; HC: healthy controls; UPDRS: unified Parkinson’s disease rating scale; H&Y, Hoehn and Yahr disability scale; FWHM, full width at half maximum; NA, not available; AFNI, Analysis of Functional Neuroimage software; SPM, statistical parametric mapping; DPARSF, Data Processing Assistant for Resting-State fMRI software; DPABI: Data Processing & Analysis for Brain Imaging; GRF: Gaussian random field; a: PD with depression; b: PD without depression; c: Tremor-dominant PD; d: Postural instability and gait difficulty PD; e: tremor-dominant PD with sleep disorder; f: tremor-dominant PD with no sleep disorder; g: PD with restless legs syndrome; h: PD without restless legs syndrome; i: PD with freezing of gait; j: PD without freezing of gait; k: PD with mild cognitive impairment; l: PD with normal cognition; m: Early-onset PD; n: Late-onset PD; o: Young HC; p: Old HC; q: PD with apathy; r: PD without apathy; s: PD with anxiety; t: PD without anxiety; u: PD with rapid eye movement sleep behavior disorder (RBD); v: PD without RBD; w: PD rs162009_A carriers; x: PD rs162009_A noncarriers.

**Reference**

Borroni, B., Premi, E., Formenti, A., Turrone, R., Alberici, A., Cottini, E., et al., (2015). Structural and functional imaging study in dementia with Lewy bodies and Parkinson's disease dementia. *Parkinsonism and Related Disorders* 21, 1049-1055.doi:10.1016/j.parkreldis.2015.06.013

Cao, Z., Yu, Y.Q., Chen, X.W., Wang, S.P., (2021). Resting state functional MRI observation on regional homogeneity and functional connectivity in Parkinson disease patients with fatigue. *Chinese Journal of Medical Imaging Technology* 37, 1814-1818.doi:10.13929/j.issn.1003-3289.2021.12.014

Chen, X., Hou, X., Luo, X., Zhou, S., Liu, X., Liu, B., et al., (2019). Altered Intra- and Inter-regional Functional Connectivity of the Anterior Cingulate Gyrus in Patients With Tremor-Dominant Parkinson's Disease Complicated With Sleep Disorder. *Front Aging Neurosci* 11, 319.doi:10.3389/fnagi.2019.00319

Choe, I.H., Yeo, S., Chung, K.C., Kim, S.H., Lim, S., (2013). Decreased and increased cerebral regional homogeneity in early Parkinson's disease. *Brain Res* 1527, 230-237.doi:10.1016/j.brainres.2013.06.027

Dai, W., Li, Z., Lin, H., Kuang, Y., Mao, H., Gan, T., et al., (2025). Resting-State Functional MRI Regional Homogeneity Correlates With Motor Scores in Parkinson's Disease. *Journal of Neuroimaging* 35.doi:10.1111/jon.70020

Feng, J.Y., Huang, B., Zhong, X.L., Yang, W.Q., Mai, F.Z., (2015). A resting-state functional MRI study based on regional homogeneity in Parkinson's disease. *Chinese Journal of Clinical Medical Imaging* 26, 844-847

Gong, T., Tao, N., Zeng, Y., Luo, W.M., Yuan, W., Wang, Y.H., et al., (2024). Regional Homogeneity Study of Resting State Functional Magnetic Resonance in Patients with Postural Instability/Gait Disorder Parkinson’s Disease. *Journal of Yichun University* 46, 52-56+75.doi:10.3969/j.issn.1671-380X.2024.06.012

Guo, W., Jin, W., Li, N., Gao, J., Wang, J., Chang, Y., et al., (2021). Brain activity alterations in patients with Parkinson's disease with cognitive impairment based on resting-state functional MRI. *Neuroscience Letters* 747.doi:10.1016/j.neulet.2021.135672

Harrington, D.L., Shen, Q., Castillo, G.N., Vincent Filoteo, J., Litvan, I., Takahashi, C., et al., (2017). Aberrant intrinsic activity and connectivity in cognitively normal Parkinson's disease. *Frontiers in Aging Neuroscience* 9.doi:10.3389/fnagi.2017.00197

Huang, Z., Wang, Y., Liu, Y., Zhang, X., Xu, D., Yang, Y., et al., (2024). Correlations between brain function and olfactory function in patients with cerebral small vessel disease and Parkinson’s disease based on resting-state functional magnetic resonance imaging. *Chinese Journal of Tissue Engineering Research* 28, 3209-3216.doi:10.12307/2024.345

Jiang, M., Fang, Y., Dai, S., Si, X., Wang, Z., Tang, J., et al., (2023). The effects of AQP4 rs162009 on resting-state brain activity in Parkinson's disease. *CNS Neurosci Ther* 29, 2645-2655.doi:10.1111/cns.14208

Jiang, X., Pan, Y., Zhu, S., Wang, Y.X., Gu, R.X., Jiang, Y.Y., et al., (2022). Alterations of Regional Homogeneity in Parkinson's Disease with Rapid Eye Movement Sleep Behavior Disorder. *NEUROPSYCHIATRIC DISEASE AND TREATMENT* 18, 2967-2978.doi:10.2147/NDT.S384752

Lan, Y., Liu, X., Yin, C., Lyu, J., Xiaoxaio, M., Cui, Z., et al., (2023). Resting-state functional magnetic resonance imaging study comparing tremor-dominant and postural instability/gait difficulty subtypes of Parkinson's disease. *Radiol Med* 128, 1138-1147.doi:10.1007/s11547-023-01673-y

Li, J., Liao, H., Wang, T., Zi, Y., Zhang, L., Wang, M., et al., (2021). Alterations of Regional Homogeneity in the Mild and Moderate Stages of Parkinson's Disease. *Front Aging Neurosci* 13, 676899.doi:10.3389/fnagi.2021.676899

Li, J.Y., Lu, Z.J., Suo, X.L., Li, N.N., Lei, D., Wang, L., et al., (2020a). Patterns of intrinsic brain activity in essential tremor with resting tremor and tremor-dominant Parkinson’s disease. *Brain Imaging and Behavior* 14, 2606-2617.doi:10.1007/s11682-019-00214-4

Li, K., Tian, Y., Chen, H., Ma, X., Li, S., Li, C., et al., (2023). Temporal Dynamic Alterations of Regional Homogeneity in Parkinson's Disease: A Resting-State fMRI Study. *Biomolecules* 13.doi:10.3390/biom13060888

Li, M.G., Liu, T.F., Zhang, T.H., Chen, Z.Y., Nie, B.B., Lou, X., et al., (2020b). Alterations of regional homogeneity in Parkinson’s disease with mild cognitive impairment: a preliminary resting-state fMRI study. *Neuroradiology* 62, 327-334.doi:10.1007/s00234-019-02333-7

Li, Y., Liang, P., Jia, X., Li, K., (2016). Abnormal regional homogeneity in Parkinson's disease: A resting state fMRI study. *Clinical Radiology* 71, e28-e34.doi:10.1016/j.crad.2015.10.006

Li, Z., Chen, J., Lin, Y., Zhou, M., Cai, Q., Li, X., et al., (2019). Reduced regional activity and functional connectivity within sensorimotor network in Parkinson's patients with restless legs syndrome. *Mol Pain* 15, 1744806919882272.doi:10.1177/1744806919882272

Liu, H., Fan, G.G., Xu, K., Shang, X.L., He, Z.Y., (2011). Alterations in regional homogeneity of resting state brain activity in Parkinson disease. *Chinese Journal of Medical Imaging Technology* 27, 1967-1971.doi:10.13929/j.1003-3289.2011.10.040

Liu, H.H., Jiang, Y.C., Liu, Y.C., Wang, Y., Qiao, L.L., (2020a). Study of alertness function resting -state magnetic resonance imaging in patients with Parkinson′s disease. *Chinese Journal of Medical Guide* 17, 177-180,196

Liu, J.X., Gan, J., Ji, L., (2020b). Resting-state fMRI study on early Parkinson’s disease with olfactory dysfunction. *Chinese Journal of Integrative Medicine and Medical Imaging* 18, 342-346.doi:10.3969/j.issn.1672-0512.2020.04.007

Liu, X., Zheng, J.Z., Liu, B., Chen, J., Li, N.N., (2012). Functional Magnetic Resonance Imaging of Regional Homogeneity Changes in Parkinsonian Resting Tremor. *Journal of Clinical Radiology* 31, 488-491.doi:10.13437/j.cnki.jcr.2012.04.005

Liu, Y., Li, M., Chen, H., Wei, X., Hu, G., Yu, S., et al., (2019). Alterations of Regional Homogeneity in Parkinson's Disease Patients With Freezing of Gait: A Resting-State fMRI Study. *Front Aging Neurosci* 11, 276.doi:10.3389/fnagi.2019.00276

Lu, Y., Li, Y., Yang, W.R., Yang, L., Zhang, H.Y., Xu, Y., et al., (2022). Dynamic changes of regional homogeneity in patients with Parkinson's disease based on resting-state functional magnetic resonance imaging. *Magnetic Resonance Imaging* 13, 45-49.doi:10.12015/issn.1674-8034.2022.06.009

Luo, B., Lu, Y., Qiu, C., Dong, W., Xue, C., Liu, D., et al., (2021). Altered Regional Homogeneity and Functional Connectivity during Microlesion Period after Deep Brain Stimulation in Parkinson's Disease. *Parkinsons Dis* 2021, 2711365.doi:10.1155/2021/2711365

Ma, W.Y., Yao, Q., Xiao, C.Y., Huang, Q.L., Shi, J.P., (2018). Alterations of dementia with Lewy body and Parkinson’s disease：a multimodal fMRI study. *Journal of Nanjing Medical University (Natural Science Edition)* 38, 1286-1291.doi:10.7655/nydxbns20180922

Oh, S.W., Shin, N.Y., Yoon, U., Sin, I., Lee, S.K., (2020). Shared functional neural substrates in Parkinson's disease and drug-induced parkinsonism: association with dopaminergic depletion. *Sci Rep* 10, 11617.doi:10.1038/s41598-020-68514-0

Peng, Q.J., Wang, Y.F., Tan, J., Hou, C.Y., Wang, Y.X., Wang, X.M., (2020). Study of fMRI of brain networks of patients with Parkinson's disease accompanied by cognition impairment *Journal of Alzheimer's Disease and Related Disorders* 3, 267-273+276+257.doi:10.3969/j.issn.2096-5516.2020.04.002

Qiu, Y.H., Nie, K., Gao, Y.Y., Wang, W.Y., Xu, Q.H., Zhang, Y.H., et al., (2020). Regional homogeneity in the patients of Parkinson disease with depression：a resting-state functional magnetic resonance imaging study *Chinese Journal of Neurology and Psychiatry* 46, 202-209.doi:10.3969/j.issn.1002-0152.2020.04.003

Sheng, K., Fang, W., Su, M., Li, R., Zou, D., Han, Y., et al., (2014). Altered spontaneous brain activity in patients with Parkinson's disease accompanied by depressive symptoms, as revealed by regional homogeneity and functional connectivity in the prefrontal-limbic system. *PLoS One* 9, e84705.doi:10.1371/journal.pone.0084705

Sun, H.H., Pan, P.L., Hu, J.B., Chen, J., Wang, X.Y., Liu, C.F., (2020). Alterations of regional homogeneity in Parkinson's disease with "pure" apathy: A resting-state fMRI study. *J Affect Disord* 274, 792-798.doi:10.1016/j.jad.2020.05.145

Sun, J.N., Yan, L., Liu, W.G., Xie, C.M., Hu, X., (2018). Alterations in regional homogeneity of resting state brain activity in Parkinson′s disease patients with depression. *Chinese Journal of Neurology* 51, 492-497.doi:10.3760/cma.j.issn.1006-7876.2018.07.003

Wang, F., Li, X.L., Cao, D.N., Fan, Y., Qu, B., Zhao, G.J., et al., (2014). Brain acticity of Parkinson's disease patients based on resting state fMRI. *Chinese Journal of Gerontology*, 5333-5335.doi:10.3969/j.issn.1005-9202.2014.19.001

Wang, M., Wang, J.W., Zhang, K.Z., Wang, D.H., Wang, J., (2019). Alterations of brain activity in different motor subtypes of Parkinson disease based on regional homogeneity analysis. *Chinese Journal of Radiology* 53, 748-754.doi:10.3760/cma.j.issn.1005-1201.2019.09.007

Wang, X., Shen, Y., Wei, W., Bai, Y., Li, P., Ding, K., et al., (2024). Alterations of regional homogeneity and functional connectivity in different hoehn and yahr stages of Parkinson's disease. *Brain Res Bull* 218, 111110.doi:10.1016/j.brainresbull.2024.111110

Wang, X., Wei, W., Bai, Y., Shen, Y., Zhang, G., Ma, H., et al., (2023). Intrinsic brain activity alterations in patients with Parkinson's disease. *Neurosci Lett* 809, 137298.doi:10.1016/j.neulet.2023.137298

Wang, Y., Zhang, S., Yang, H., Zhang, X., He, S., Wang, J., et al., (2021). Altered cerebellum functional network on newly diagnosed drug-naïve Parkinson's disease patients with anxiety. *Transl Neurosci* 12, 415-424.doi:10.1515/tnsci-2020-0192

Wen, Z., Zhang, J., Li, J., Dai, J., Lin, F., Wu, G., (2016). Altered Activation in Cerebellum Contralateral to Unilateral Thalamotomy May Mediate Tremor Suppression in Parkinson's Disease: A Short-Term Regional Homogeneity fMRI Study. *PLoS One* 11, e0157562.doi:10.1371/journal.pone.0157562

Wu, T., Long, X., Zang, Y., Wang, L., Hallett, M., Li, K., et al., (2009). Regional homogeneity changes in patients with Parkinson's disease. *Hum Brain Mapp* 30, 1502-1510.doi:10.1002/hbm.20622

Xian Liu, B.L., Jun Chen, Zhiguang Chen Department of Radiology, Guangdong Province Traditional Chinese Medicine Hospital, Guangzhou 510120, Guangdong Province, China, (2011). Functional magnetic resonance imaging of regional homogeneity changes in parkinsonian resting tremor. *Neural Regeneration Research* 6, 811-815.doi:10.3969/j.issn.1673-5374.2011.11.002

Xing, Y., Fu, S., Li, M., Ma, X., Liu, M., Liu, X., et al., (2021). Regional Neural Activity Changes in Parkinson's Disease-Associated Mild Cognitive Impairment and Cognitively Normal Patients. *Neuropsychiatr Dis Treat* 17, 2697-2706.doi:10.2147/ndt.S323127

Xu, H., Zhang, M., Wang, Z., Yang, Y., Chang, Y., Liu, L., (2022). Abnormal brain activities in multiple frequency bands in Parkinson's disease with apathy. *Front Neurosci* 16, 975189.doi:10.3389/fnins.2022.975189

Yang, H., Zhou, X.J., Zhang, M.M., Zheng, X.N., Zhao, Y.L., Wang, J., (2013). Changes in spontaneous brain activity in early Parkinson's disease. *Neuroscience Letters* 549, 24-28.doi:10.1016/j.neulet.2013.05.080

Yeo, S., Lim, S., Choe, I.H., Choi, Y.G., Chung, K.C., Jahng, G.H., et al., (2012). Acupuncture stimulation on gb34 activates neural responses associated with parkinson's disease. *CNS Neuroscience and Therapeutics* 18, 781-790.doi:10.1111/j.1755-5949.2012.00363.x

Yue, Y., Jiang, Y., Shen, T., Pu, J., Lai, H.Y., Zhang, B., (2020). ALFF and ReHo Mapping Reveals Different Functional Patterns in Early- and Late-Onset Parkinson's Disease. *Front Neurosci* 14, 141.doi:10.3389/fnins.2020.00141

Zang, Z.X., Song, T.B., Cui, B.X., Yang, H.W., Zhang, C., Lu, J., (2023). Integrated PET/MRI for evaluating association between cerebral glucose metabolism and regional homogeneity in patients with Parkinson's disease. *Chinese Journal of Medical Imaging Technology* 39, 1467-1471.doi:10.13929/j.issn.1003-3289.2023.10.006

Zhang, J., Wei, L., Hu, X., Xie, B., Zhang, Y., Wu, G.R., et al., (2015). Akinetic-rigid and tremor-dominant Parkinson's disease patients show different patterns of intrinsic brain activity. *Parkinsonism and Related Disorders* 21, 23-30.doi:10.1016/j.parkreldis.2014.10.017

Zhang, P., Zhang, Y., Luo, Y., Wang, L., Wang, K., (2022). Regional activity alterations in Parkinson's disease patients with anxiety disorders: A resting-state functional magnetic resonance imaging study. *Front Aging Neurosci* 14, 1055160.doi:10.3389/fnagi.2022.1055160

Zhong, Y.H., Zhang, R., Ge, H.T., Zhu, H., Gong, P., (2018). Classification of Parkinson's disease based on abnormal brain region voxels with rest-stating functional magnetic resonance imaging. *Journal of Xuzhou Medical University* 38, 21-25.doi:10.3969/j.issn.1000-2065.2018.01.006

Zhu, H., Zhu, H., Liu, X., Zhou, Y., Wu, S., Wei, F., et al., (2022). Alterations of Regional Homogeneity in Parkinson's Disease: A Resting-State Functional Magnetic Resonance Imaging (fMRI) Study. *Cureus* 14, e26797.doi:10.7759/cureus.26797

Zhu, Q.R., Zhou, M.L., Li, D., Fu, L.D., Hu, Z.X., Lu, X.D., (2020). Alterations in regional homogeneity on resting-state fMRI in Parkinson's disease. *Zhejiang Medical Journal* 42, 357-360+411.doi:10.12056/j.issn.1006-2785.2020.42.4.2018-1665
